# Unlocking the potential of bacterial endophytes from medicinal plants for drug discovery

**DOI:** 10.1111/1751-7915.14382

**Published:** 2024-02-12

**Authors:** Sergey B. Zotchev

**Affiliations:** ^1^ Division of Pharmacognosy, Department of Pharmaceutical Sciences University of Vienna Vienna Austria

## Abstract

Among the plant‐associated microorganisms, the so‐called endophytes continue to attract much attention because of their ability not only to protect host plants from biotic and abiotic stress factors, but also the potential to produce bioactive secondary metabolites. The latter property can elicit growth‐promoting effects on plants, as well as boost the production of plant‐specific secondary metabolites with valuable pharmacological properties. In addition, endophyte‐derived secondary metabolites may be a rich source for the discovery of drugs to treat various diseases, including infections and cancer. However, the full potential of endophytes to produce bioactive secondary metabolites is often not revealed upon conventional cultivation in the laboratory. New advances in genomics and metabolic engineering offer exciting opportunities for the exploration and exploitation of endophytes' biosynthetic potential. This review focuses on bacterial endophytes of medicinal plants, some of their secondary metabolites and recent advances in deciphering their biosynthesis. The latter may assist in genetic engineering efforts aimed at the discovery of novel bioactive compounds with the potential to be developed into drugs.

## Graphical abstract

Bacteria dwelling within tissues of medicinal plants, the so‐called endophytes, represent a unique source of secondary metabolites with diverse biological activities. Modern approaches such as “genome mining” and metabolic engineering provide new opportunities for revealing the biosynthetic potential of bacterial endophytes and assisting in identification of novel compounds which may fuel drug discovery pipelines.

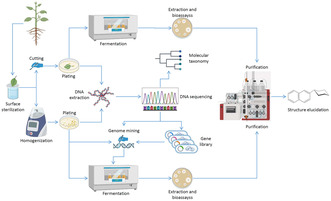

## INTRODUCTION

Medicinal plants have been used for thousands of years in traditional medicine to treat various human diseases, including infections and cancer, as well as for the alleviation of related symptoms (Atanasov et al., 2021). Up until the 20th century, most of the healing properties of medicinal plants have been fulfilled via plant extracts, usually prepared as ethanol or water infusions. Although in many cases the medicinal effect of plant extracts was most likely due to a combination of bioactive natural products in these very complex mixtures, the presence of harmful substances could not be ruled out. In addition, variations in growth conditions, such as temperature, soil composition, humidity, UV exposure etc. most likely affect the ratio of bioactive compounds in plant extracts. To circumvent these problems, later efforts have focused on the identification of individual bioactive components in complex plant extracts which could be used as controlled, single‐component medicines, such as for example, paclitaxel, an anti‐cancer drug from *Taxus brevifolia* and artemisinin, an anti‐malarial drug from *Artemisia annua*. At the same time, a large number of rigorously controlled and tested plant extracts are continued to be used as medicines, apparently due to the synergistic healing effects of their components (Atanasov et al., 2015).

It has long since been noted, that the same medicinal plant collected from different geographic locations may have quite different extract compositions (Liu et al., 2018). While this can in part be explained by differences in soil composition and climate, recent insights into microbiomes of medicinal plants suggest that endophytes, microorganisms dwelling within plant tissues while causing no harm to the host, may have a significant effect on the ability of plants to produce specific bioactive compounds. Several in vitro studies have demonstrated that endophytic fungi and bacteria can be used as elicitors of plant‐specific secondary metabolite production in plant tissue cultures (Khare et al., 2018; Taghinasab & Jabaji, 2020). Moreover, certain endophytes were shown to exert beneficial effects on plants, including growth promotion and defence against biotic and abiotic stresses (Chiaranunt & White, 2023). Better understanding of the microbial ecology of plants and their associated endophytes may prove important for the discovery and development of new and effective human drugs.

In the past few decades, a number of bacterial endophytes have been isolated from a variety of plants, including those used in traditional medicine, and some of them were shown to produce unique bioactive secondary metabolites (Igarashi, 2023). The latter compounds are natural products that are not essential for a basic functioning of bacterial cells in the laboratory, but may give their producers an advantage in the natural environment. For example, secondary metabolites may have a role as chemical weapons to repel predators, suppress competitors for nutritional sources (Spagnolo et al., 2021), or serve as metal chelators or signalling molecules for communication with other members of microbial community living within the plant (Bradley et al., 2020; Scherlach & Hertweck, 2020). Interestingly, the ecological functions of secondary metabolites produced by plants appear to be somewhat different from those produced by endophytic bacteria. Indeed, some plant‐specific secondary metabolites are known to function as signal molecules used by the plants to attract pollinating insects or seed‐dispersing animals (Wink, 2003). Secondary metabolites are biosynthesized by both plants and microorganisms via sophisticated pathways involving various enzymes and precursors derived from primary metabolism. Several studies have shown that bacterial endophytes can produce bioactive secondary metabolites that are similar or even identical to those isolated from host plants (Ludwig‐Müller, 2015). In addition, a number of unique secondary metabolites never detected in the host plant extracts have been isolated from endophytic bacteria of medicinal plants. Hence, continued bioprospecting of bacterial endophytes for novel bioactive secondary metabolites along with advances in understanding of their biosynthesis may have a significant impact on drug discovery.

## MEDICINAL PLANTS AND ASSOCIATED MICROORGANISMS

It is widely acknowledged that plants acquire most of their endophytes from the rhizosphere (Compant et al., 2021), although some studies have also demonstrated the direct transfer of endophytes via seeds (Johnston‐Monje et al., 2022). Plant roots extrude into soil substances such as cellulose, organic, amino and fatty acids, phenolics, plant growth hormones, nucleotides, sugars, sterols and vitamins that attract microorganisms and can be used by them as nutritional sources, (Sasse et al., 2018). Bacteria which have the capacity for chemotaxis, the ability to sense specific molecules and move toward or away from their source (Kumar et al., 2020), have an advantage in colonizing the plants by intruding through the root tips or tiny lesions in the roots (Acar et al., 2022). Some bacteria were shown to invade roots using enzymes such as cellulases, xylanases and endoglucanases, which are capable of degrading plant cell wall (Liu et al., 2017). Other, non‐motile microorganisms such as fungi and filamentous bacteria (e.g. of the genus *Streptomyces*) that are often reproduced via spores, can colonize the surface of the root first before being able to invade plant tissues. More information on the acquisition of endophytic bacteria by plants can also be found in the following reviews (Hardoim et al., 2015; Oukala et al., 2021; Wassermann et al., 2022).

The effect of bacterial endophytes on the fitness of medicinal plants and their capacity to produce plant‐specific secondary metabolites is well documented (Afzal et al., 2019; Oukala et al., 2021). For example, endophytic bacteria from *Thymus vulgaris* were shown to both alleviate the abiotic stress (salinity) of host plants and to protect them from a fungal pathogen (Abdelshafy Mohamad et al., 2020). More importantly, certain endophytic bacteria can stimulate the production of medicinally useful plant products. It has been shown that endophytic *Acinetobacter* sp. isolated from the opium poppy *Papaver somniferum* L. stimulates expression of the genes involved in the biosynthesis of benzylisoquinoline alkaloids in this plant (Pandey et al., 2016). *Pseudomonas fluorescens* ALEB7B, an endophyte of the medicinal plant *Atractylodes lancea* traditionally used in Chinese herbal medicine, was shown to stimulate the production of oxygenous sesquiterpenoids, the main bioactive components of the plant (Zhou et al., 2018). The beneficial properties of bacterial endophytes that stimulate the production of plant‐specific metabolites are, however, difficult to exploit in a sustainable way. Treatment of medicinal plants with endophytic bacteria in the fields appears problematic both from technical and environmental points of view and stimulation of plant tissue or suspension cultures in bioreactors on a large scale is yet to be demonstrated (Wawrosch & Zotchev, 2021). Hence, the exploration of endophytic bacteria for novel bioactive secondary metabolites and their biosynthetic pathways appears a more attractive path to the discovery of drugs and their sustainable production.

## BACTERIAL ENDOPHYTES OF MEDICINAL PLANTS AND THEIR SECONDARY METABOLITES

A survey of the literature (PubMed) published since 2018 and up until April 2023 on the topics of endophytes from medicinal plants identified 690 articles, more than 90% of which were dedicated to fungal endophytes and secondary metabolites therefrom. This clear bias may come from the traditional approach of focusing on the isolation of endophytic fungi used for many decades. The absolute majority of the articles on bacterial endophytes of medicinal plants described the biological activity of culture extracts of these bacteria, with only a few reporting the identification of secondary metabolites. The latter may be explained by the poor performance of bacterial endophytes under laboratory conditions in terms of secondary metabolite production, possibly caused by the absence of plant‐specific factors triggering the expression of the genes governing the biosynthetic pathways.

**FIGURE 1:**
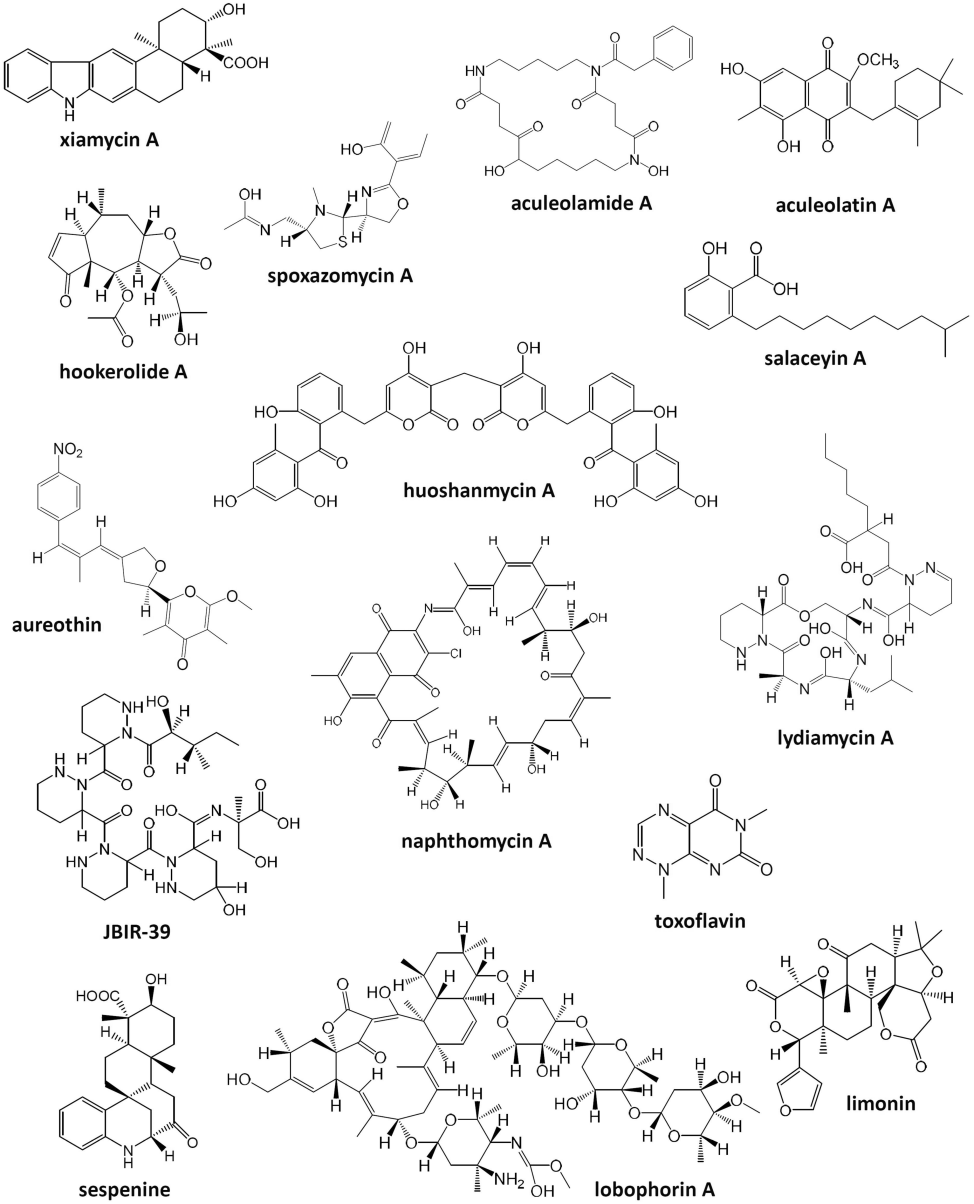
Structural diversity of secondary metabolites isolated from endophytic bacteria of medicinal plants.

A rather common theme in the research on endophytes is an attempt to connect their ability to synthesize particular bioactive compounds and the medicinal use of their host plant. Although in some cases this may indeed be relevant (e.g. for lobophorins, see below), it seems rather unlikely that biosynthetic abilities of endophytic bacteria cultivated in the laboratory are exhibited in the same way as in planta. Below are descriptions of several secondary metabolites isolated from endophytic bacteria, whose bioactivities do not always correlate with the medicinal use of their host plants (Figure 1; Table 1), but may represent unique starting points for drug discovery.

**TABLE 1:** Examples of bioactive secondary metabolites produced by endophytic bacteria isolated from medicinal plants.

Compound	Chemical class	Biological activity	Producer endophyte	Endophyte source	Traditional use of the host plant	Reference
Piericidins	Monohydroxypyridines	Anti‐bacterial, cytotoxic	*Streptomyces* sp. *KIB‐H1083*	*Diphasiastrum veitchii*	Anti‐inflammatory	Shang et al. (2018)
Salaceyins	Salicylates	Anti‐fungal, cytotoxic	*Streptomyces laceyi*	*Ricinus communis L*.	Abdominal disorders, arthritis, backache, muscle aches	Kim et al. (2006)
Aureothin	Polyethers	Nematicidal	*Streptomyces* sp. *AE170020*	*Pinus densiflora*	Kidney and bladder problems, treatment of rheumatic symptoms	Kang et al. (2022)
Aculeolatins	Meroterpenoids	Anti‐bacterial, anti‐malarial	*Streptomyces aculeolatus MS1‐6*	*Musa sapientum* Linn. (ABB group) cv. ‘Kluai Namwa’	Anti‐inflammatory and antioxidant	Kuncharoen et al. (2023)
Aculeolamides	Hydroxamate siderophores	Anti‐bacterial, anti‐malarial	*Streptomyces aculeolatus MS1‐6*	*Musa sapientum* Linn. (ABB group) cv. ‘Kluai Namwa’	Anti‐inflammatory and antioxidant	Kuncharoen et al. (2023)
Lobophorins	Spirotetronate macrolides	Anti‐inflammatory, cytotoxic	*Streptomyces olivaceus* JB1	*Maesa japonica* (Thunb.) Moritzi & Zoll.	Remedy against fever and hepatitis	Um et al. (2022)
Limonin	Triterpenes	Neuroprotective	*Bacillus* sp. P	*Citrus maxima* (Burm.) Merr. cv. Shatian Yu.	Treatment of cough, fever, asthma, diarrhoea, ulcer	Zhang, Fan, et al. (2019); Zhang, Zang, et al. (2019)
JBIR‐39	Cyclic peptides	Anti‐bacterial	*Streptomyces* sp. AB100	*Atropa belladonna* (L.)	Pain reliever, anti‐inflammatory, anti‐motion sickness	Bekiesch et al. (2021)
Sespenins	Indolosesquiterpenes	Anti‐bacetrial, cytotoxic	*Streptomyces* sp. HKI0595	*Kandelia candel*	Diuretic and laxative	Ding et al. (2011)
Xiamycins	Indolosesquiterpenes	Anti‐HIV activity	*Streptomyces* sp. GT2002/1503	*Bruguiera gymnorrhiza*	Treatment of ulcerative colitis	Ding et al. (2010)
Fusaricidins	Depsipeptides	Anti‐fungal	*Paenibacillus polymyxa Y‐1*	*Dendrobium nobile*	Tonic, astringent, analgesic, antipyretic, anti‐inflammatory	Yang, Yang, et al. (2018); Yang, Zeng, et al. (2018)
Munumbicins	Lipopeptides	Anti‐bacterial, anti‐fungal	*Streptomyces* sp. *NRRL 30562*	*Kennedia nigriscans*	Healing of skin wounds and infections	Castillo et al. (2002)
Coronamycin	Lipopeptides	Anti‐fungal, anti‐malarial	*Streptomyces* sp. MSU‐2110	*Monstera* sp.	Treatment of arthritis and insect bites	Ezra et al. (2004)
Huoshanmycins	Polyketides	Cytotoxic	*Streptomyces* sp. HS‐3‐L‐1	*Dendrobium huoshanense*	Tonic, astringent, analgesic, antipyretic, anti‐inflammatory	Zhu et al. (2022)
Lydiamycins A, E‐H	Cyclodepsipeptides	Anti‐mycobacterial, antimetastatic	*Streptomyces* sp. HBQ95	*Cinnamomum cassia* Presl	Anti‐tumour, anti‐inflammatory, analgesic, cytoprotective	Wang et al. (2023)
Mojavensin A	Lipopeptides	Anti‐fungal, cytotoxic	*Bacillus halotolerans* Cal.l.30	*Calendula officinalis*	Treatment of burns, bruises, cuts and minor infections	Tsalgatidou et al. (2022)
Eudesmane‐5β,11‐diol	Polycyclic sesquiterpenes	Anti‐bacterial, anti‐fungal	*Streptomyces* sp. JMRC:ST027706	*Bruguiera gymnorrhiza*	Diarrhoea, fever, diabetes, pain	Ding and Hertweck (2020)
Chartspiroton	Spiro‐naphthoquinones	Non reported	*Streptomyces* sp. SH‐1.2‐R‐15	*Dendrobium officinale*	Tonic, astringent, analgesic, antipyretic, anti‐inflammatory	Zhao, Yang, Liu, et al. (2020)
Endostemonines	Pyrrole‐2‐carboxylic esters	Insecticidal	*Streptomyces* sp. BS‐1	*Stemona sessilifolia*	Remedy for parasitic infections	Zhao, Yang, Zhang, et al. (2020)
Toxoflavin	Pyrimidotriazines	Anti‐fungal	*Burkholderia gladioli* HDXY‐02	*Lycoris aurea*	Treatment of swellings, ulcers, nervous afflictions of children	Li et al. (2019)
Tetramethylpyrazine	Alkylpyrazines	Anti‐inflamatory, nootropic	*Bacillus subtilis* LB5	*Ligusticum chuanxiong* Hort.	Promoting blood circulation and removing blood clots	Yin et al. (2018)
Naphthomycins	Naphthoquinones	Cytotoxic	*Streptomyces* sp. CS	*Maytenus hookeri*	Treatment of gastric ulcers and skin allergies	Yang, Yang, et al. (2018); Yang, Zeng, et al. (2018)
Hookerolides	Pseudoguaianolides	Cytotoxic	*Streptomyces* sp. CS	*Maytenus hookeri*	Treatment of gastric ulcers and skin allergies	Yang, Yang, et al. (2018); Yang, Zeng, et al. (2018)

Polyether aureothin has been first characterized as a cytotoxic compound, originally isolated from *Streptomyces thioluteus* (Washizu et al., 1954). Later, aureothin and its congeners were identified in several *Streptomyces* bacteria, including *Streptomyces* sp. AE170020, an endophyte of *Pinus densiflora* (Japanese red pine). Extracts from this plant are traditionally used to treat kidney and bladder disorders, as well as rheumatic symptoms. Aureothin was shown to have potent nematicidal and anti‐trypanosomal activities (Kang et al., 2022; Otoguro et al., 2008), while the exact mechanism of action of this compound remains to be elucidated. Unusual structural features of aureothin triggered considerable interest as to how it is biosynthesized and whether it is possible to generate new aureothin analogues (see sections below).

Endophytic actinomycete bacterium *Streptomyces aculeolatum* MS1‐6 isolated from the medicinal plant *Musa sapientum* (dessert banana, known for anti‐inflammatory and anti‐oxidant activities) was shown to produce a series of compounds with anti‐bacterial and anti‐malarial activities (Kuncharoen et al., 2023). Six congeners of new naphthoquinone terpenoids aculeolatins were identified, and three of those exhibited significant anti‐bacterial and anti‐malarial activities while having low cytotoxicity. One of the two congeners of hydroxamate siderophores aculeolamides identified in this study was active against *Mycobacterium tuberculosis* and *Plasmodium falciparum* but had only negligible cytotoxicity.

Um et al. (2022) recently described the isolation of several congeners of the spirotetronate macrolide lobophorin from *Streptomyces olivaceus* JB1, an endophytic actinomycete bacterium from *Maesa japonica* (broad flat‐rock tree). In this case, lobophorins could also be detected in the extracts from the dried leaves of the plant, suggesting that *S. olivaceus* JB1 could be responsible for the production of these compounds in planta. Lobophorins have been isolated earlier from a number of marine‐derived *Streptomyces*, and were shown to have anti‐microbial, anti‐inflammatory and cytotoxic activities (Jiang et al., 1999; Luo et al., 2021; Wei et al., 2011). Extracts from *Maesa japonica* are being used as a remedy against fever and in skincare, and it is possible that lobophorins produced by the abovementioned endophyte contribute to these medicinal properties of the plant.

Xiamycins and sespenins are indolosesquiterpenes of apparently common biosynthetic origin isolated in the Hertweck lab from *Streptomyces* endophytes of medicinal plants *Bruguiera gymnorrhiza* (Ding et al., 2010) and *Kandelia candel* (Ding et al., 2011). Fruits of *B. gymnorrhiza* (a medicinal mangrove tree), are used to treat diarrhoea, while extracts from *K. candel* (also a mangrove tree) have diuretic and laxative properties. It appears unlikely that xiamycins, which have selective anti‐HIV activity, and sespenine with anti‐bacterial and cytotoxic activities contribute to the medicinal properties of the host plants. Nevertheless, the discovery of these indolosesquiterpenes triggered considerable interest with regard to the antiviral activities of these compounds (Meng et al., 2015).

Cyclodepsipetides with anti‐mycobacterial activity, lydiamycins A‐D, were initially isolated from soil‐dwelling *Streptomyces lydicus* HKI0343 (Huang et al., 2006). Recently, lydiamycin A and its novel congeners, lydiamycins E‐H were identified in *Streptomyces* sp. HBQ95, an endophytic actinomycete bacterium associated with the medicinal plant *Cinnamomum cassia* Presl (Wang et al., 2023). The bark of this plant, Chinese cinnamon known for its medicinal properties, is used in traditional medicine as anti‐tumour, anti‐inflammatory and analgesic remedy. The lydiamycin congeners isolated from *Streptomyces* sp. HBQ95 were shown to have anti‐metastatic activity without having a significant cytotoxicity, which correlates with the uses of the plant. However, there have been no reports on the identification of lydiamycins in the bark of Chinese cinnamon, which contains over 160 phytochemicals (Zhang, Fan, et al., 2019; Zhang, Zang, et al., 2019).

Endophytic *Streptomyces* sp. HS‐3‐L‐1 isolated from the leaf of *Dendrobium huoshanense*, an orchid used in traditional medicine due to its tonic, analgesic and anti‐inflammatory effects, produced unique polyketide dimers huoshanmycins A–C (Zhu et al., 2022). The monomers, of which huoshanmycins are composed, apparently have biosynthetic origin analogous to that of polyketides SEK43, SEK15 or UWM4 reported previously as products of a minimal polyketide synthase (PKS) type II and a cyclase involved in the biosynthesis of cytotoxic angucycline jadomycin (Meurer et al., 1997). Hence, the monomers of huoshanmycins are likely to be biosynthesized by a similar PKS system, while being connected via methylene linkages by as of yet unknown mechanism. While biological activities of monomers have not been reported, the huoshanmycins were shown to have moderate cytotoxic activity against human cells of acute myeloid leukaemia (Zhu et al., 2022).

Toxoflavin, pyrimidotriazine antibiotic and phytotoxin, was first isolated from a strain of *Pseudomonas cocovenenans* (van Damme et al., 1960), later re‐classified as *Burkholderia gladioli* (Li et al., 2019). This bacterium originates from a medicinal plant *Lycoris aurea* (red spider lily) known to produce galantamine, an alkaloid approved by the United States Food and Drug Administration for the treatment of Alzheimer's disease. Besides being a potential source of galantamine, red spider lily extracts are used traditionally to treat swellings and ulcers. Toxoflavin, which has been isolated from several plant‐associated *Burkholderia* spp., is active against a wide range of fungi (Li et al., 2019), but is considered too toxic to find an application in medicine. Toxoflavin was shown to be the key virulence factor of phytopathogenic bacteria, for example, *Burkholderia glumae*, acting as an electron carrier that sequesters electrons from the respiratory chain of the plant and transfers them to oxygen, thereby generating toxic hydrogen peroxide (Iqbal et al., 2021). Recently published study suggested that toxoflavin can be useful as a molecular tool to study enzymes involved in sensing endoplasmic reticulum stress (Jiang et al., 2023).

The discovery of many unique secondary metabolites from endophytic bacteria prompted investigations in their biosynthesis, which unravelled novel enzyme mechanisms and provided opportunities for pathway engineering that led to the production of new bioactive analogues. Several examples of such investigations are highlighted in the next section.

## BIOSYNTHESIS OF SELECTED SECONDARY METABOLITES IN BACTERIAL ENDOPHYTES

Research on the biosynthesis of secondary metabolites in bacteria over the last four decades has unravelled hundreds of unique biochemical pathways specified by the so‐called biosynthetic gene clusters (BGCs). These are groups of co‐localized genes encoding enzymes that perform a coordinated series of reactions leading to the formation of complex chemical structures of secondary metabolites from simple building blocks originating from primary metabolism (e.g. amino acids, fatty acids, sugars, etc). The discovery and characterization of BGCs in bacteria are supported by the recent advances in efficient and low‐cost sequencing of bacterial genomes, followed by genome analyses with software specializing on the identification of BGCs and prediction of their putative products. The above‐mentioned software includes antiSMASH, ClusterFinder, NP.searcher etc (reviewed in Chavali & Rhee, 2018). These bioinformatics tools help to evaluate the potential of bacteria to biosynthesize various secondary metabolites, connect known molecules to the respective BGCs and plan experiments on the so‐called genome mining. The latter approach is aimed at activating the expression of BGCs that are otherwise ‘silent’ in the laboratory conditions either due to low transcription, problems with translation or availability of necessary precursors. Genome mining can include manipulation of transcription factors and regulatory elements controlling the expression of cognate BGCs, as well as heterologous expression of cloned BGCs in specifically engineered bacterial hosts (reviewed in Sekurova et al., 2019). Detailed bioinformatics‐assisted analyses of the secondary metabolite biosynthesis genes, accompanied by experiments designed to confirm or determine gene functions help to deduce biosynthetic pathways leading to the BGC product(s). Knowledge on such pathways can serve as a basis for metabolic and/or biosynthetic engineering intended to increase the production of the target molecule (Belcher et al., 2020), or introduce specific structural changes that may be beneficial for its pharmacological properties (Puja et al., 2023). In this section, a few examples of biosynthetic pathways for secondary metabolites identified in endophytic bacteria are discussed.

First detailed insights into the biosynthesis of toxoflavin, and the elucidation of its complete biosynthetic pathway were gained after the identification of toxoflavin BGC in the genomes of several bacteria (Philmus et al., 2015). This important achievement was later complemented by two studies that experimentally verified the functions of two genes in the toxoflavin biosynthesis pathway (Fenwick et al., 2016; Song et al., 2023), thus removing some ambiguities that were still present in the work by Philmus et al. (2015). These crucial insights into the toxoflavin biosynthesis were gained after heterologous expression of the corresponding BGC from *Burkholderia gladioli* in the heterologous host *Escherichia coli*, stressing the importance of genetic manipulations in deciphering the biosynthesis of natural products.

**FIGURE 2:**
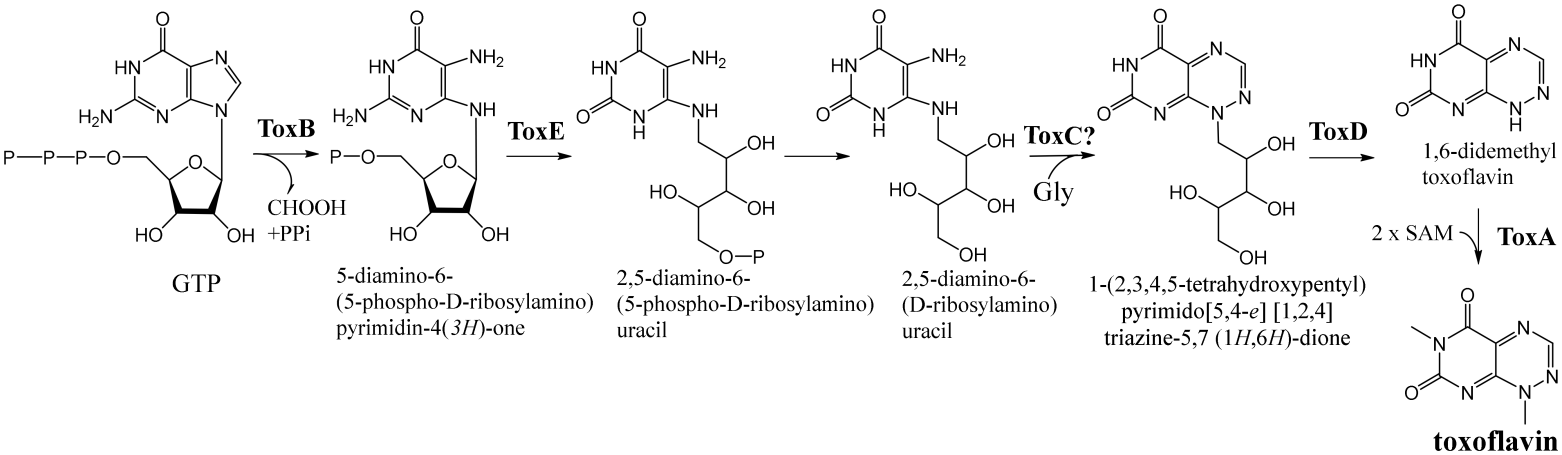
Biosynthetic pathway for toxoflavin in endophytic and phytopathogenic bacteria deduced based on the works by Philmus et al. (2015), Fenwick et al. (2016) and Song et al. (2023).

The finally emerged biosynthetic pathway leading to toxoflavin (Figure 2) starts with guanosine triphosphate, which is converted into an intermediate known from riboflavin biosynthesis, 5‐diamino‐6‐(5‐phospho‐D‐ribosylamino) pyrimidin‐4(3H)‐one, which is then deaminated. The resulting product is dephosphorylated by unspecified phosphatase and then converted into 1‐(2,3,4,5‐tetrahydroxypentyl) pyrimido[5,4‐e] [1,2,4] triazine‐5,7 (1H,6H)‐dione, which is oxidized to yield 1,6‐didemethyl toxoflavin (Song et al., 2023). Finally, the toxoflavin is generated via two subsequent N‐methylations of 1,6‐didemethyl toxoflavin (Fenwick et al., 2016).

**FIGURE 3:**
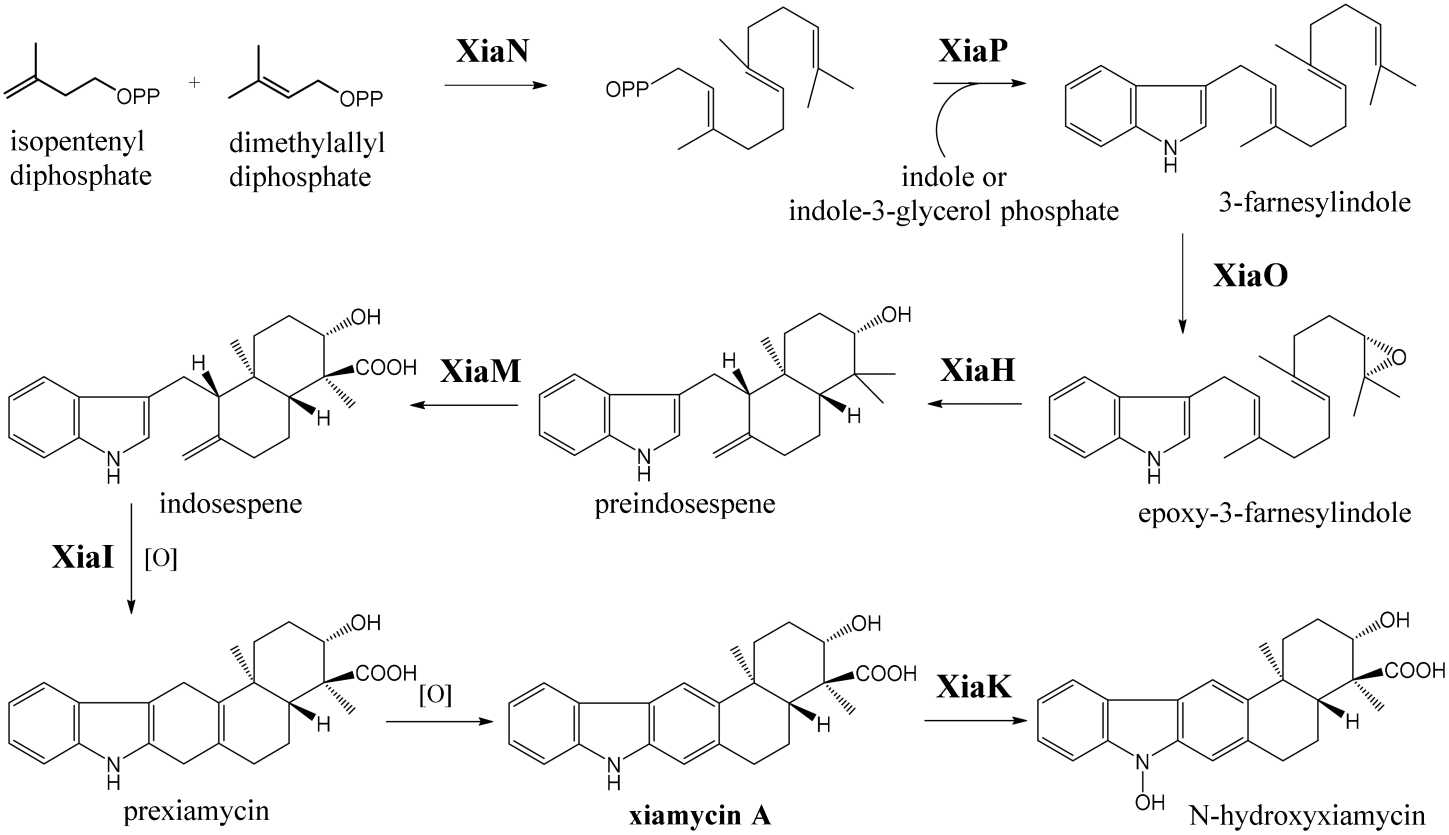
Biosynthesis of the xiamycins, indolosesquiterpenes isolated from a variety of *Streptomyces* spp., including endophytes of medicinal plants (compiled from the works of Li et al., 2012, 2015; Xu et al., 2012; Zhang et al., 2017).

Biosynthesis of the indolosesquiterpenes related to xiamycins, in particular xiamycin A (Figure 3), has been proposed by Li et al. (2012) based on the earlier study of Uchida et al. (2006) on the biosynthesis of fungal indolosesquiterpene sespendole and characterization of the xiamycin BGC in *Streptomyces* sp. SCSIO 02999. The former study has been further expanded by the elucidation of cyclization reactions leading to the formation of xiamycin pentacyclic core (Li et al., 2015) and characterization of an N‐hydroxylase responsible for chemical diversification of xiamycins (Zhang et al., 2017). The xiamycin biosynthetic pathway starts with the condensation of isopentenyl diphosphate and dimethylallyl diphosphate followed by condensation with indole‐3‐glycerol phosphate, generating 3‐farnesylindole, which is then converted to epoxy‐3‐farnesylindole. The terpene moiety of the latter is cyclized, leading to the formation of preindosespene, which is subsequently oxidized at C24 to yield indosespene. The formation of xiamycin pentacyclic core is completed by the closure of the central ring. The resulting intermediate, prexiamycin, appears to be spontaneously oxidized to xiamycin A, which can be converted to N‐hydroxyxiamycin via N‐hydroxylation. The latter modification turned out to be crucial for further structural diversification of xiamycin‐like indolosesquiterpenes to yield various xiamycin derivatives such as dixiamycins (xiamycin A, B and C dimers) and chloroxiamycin, by as yet unidentified bacterial enzymes (Zhang et al., 2017). It seems plausible that XiaK and similar N‐hydroxylases can be used for generating chemically diverse libraries of indolosesquiterpenes via biotransformation by bacterial strains expressing various enzymes, for example, halogenases.

**FIGURE 4:**
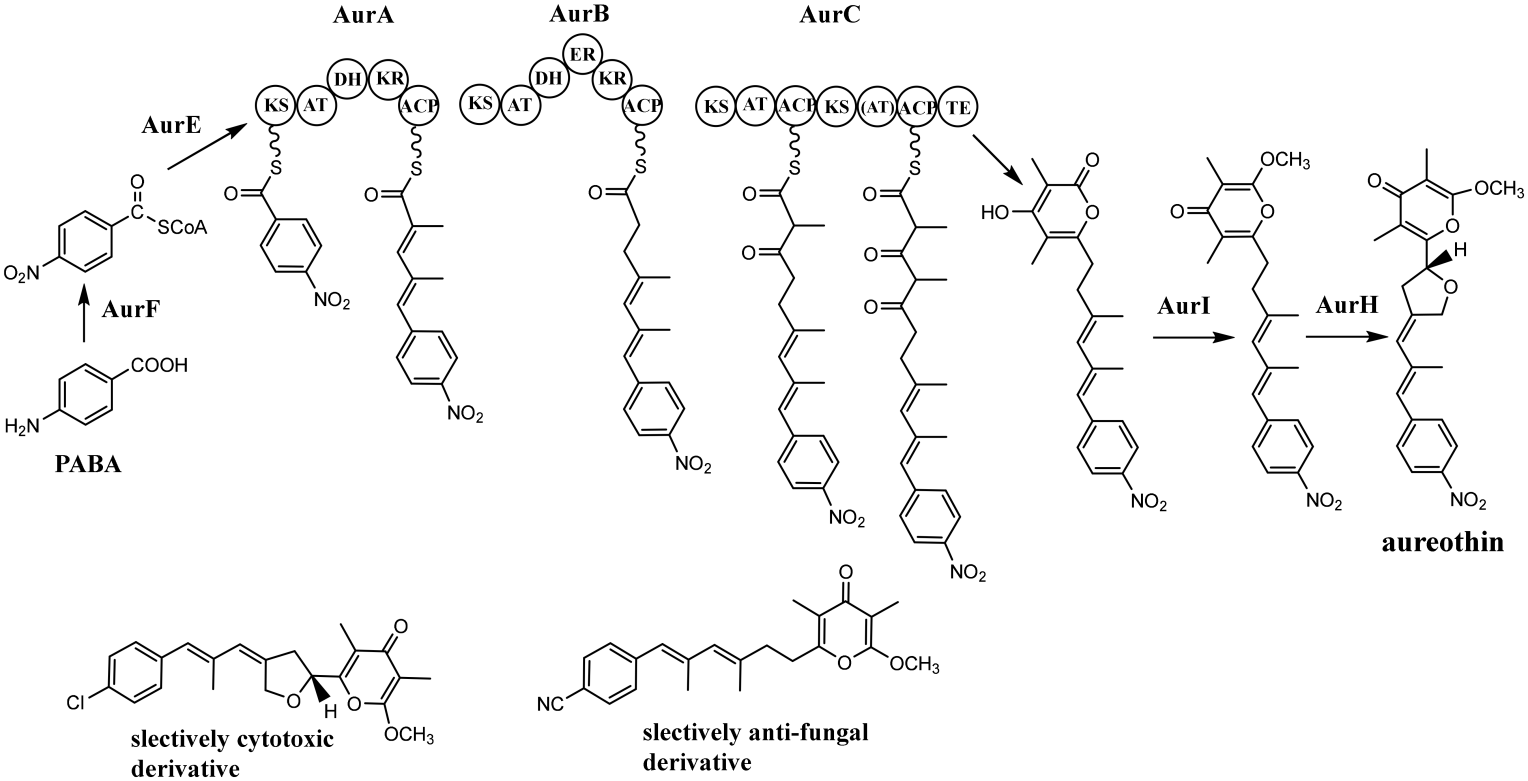
Biosynthesis of aureothin, nitrophenyl‐substituted polyketide, provided a basis of biosynthesis engineering yielding selective anti‐fungal and anti‐proliferative analogues (adopted from Werneburg et al., 2010).

First insights into the biosynthesis of aureothin (Figure 4), the nitrophenyl‐substituted polyketide with a range of biological activities, have been gained after identification, cloning and heterologous expression of the aureothin BGC from *Streptomyces thioluteus* HKI‐227 (He & Hertweck, 2003). The aureothin BGC was found to contain nine genes, of which three, *aurA*, *B* and *C*, encode type I polyketide synthases (PKS) apparently responsible for the assembly of the polyketide moiety. Polyketide synthases type I are modular enzymes, where each module is composed of several enzymatic domains responsible for the insertion of an acyl building block into the growing polyketide chain and its subsequent modification (Grininger, 2023). The first PKS type I module is called a loading module, which primes the PKS complex with particular substrates that can range from acyl units to amino acids. The extension of this starting building block is performed by the downstream modules of PKS. The choice of extender building block is governed by acyltransferase domain, which chooses a specific acyl‐CoA (typically malonyl‐ or methylmalonyl‐CoA) and tethers it to the acyl carrier protein domain. The ketosynthase domain within the module catalyses Claisen‐type decarboxylative condensation of two building blocks—one from the loading module, and another from the extender module. The acyl chain synthesized by PKS continues to grow until it reaches the final module containing the thioesterase domain, which releases the mature chain and may also assist in its cyclization. Importantly, the presence or absence of particular reductive domains within each module (ketoreductase, dehydratase, enoyl reductase) results in the modification of the polyketide by means of introduction of keto‐ or hydroxy groups, double or single bonds. Hence, PKS type I represent molecular assembly lines with defined tools that can be modified in a way that leads to predictable changes in the structure of polyketide produced by the engineered PKS (Kornfuehrer & Eustáquio, 2019).

Aureothin biosynthesis starts with the priming of AurABC PKS complex with the nitrophenyl starter, which is extended by the AurABC PKS utilizing 1 malonyl‐CoA and 4 methylmalonyl‐CoAs to assemble the polyketide backbone. He and Hertweck (2003) demonstrated that one module of the aureothin PKS acts iteratively, catalysing two successive condensations of methylmalonate building blocks, thus ensuring the extension of the nitrophenyl starter with 5 acyl units. The release of polyketide chain from the AurABC PKS is followed by the cyclization of diketoacid to form the pyrone moiety, yielding nordeoxyaureothin. In the next steps, the pyrone moiety of nordeoxyaureothin is O‐methylated, and the resulting product is processed by the cytochrome P450 enzyme AurH to form the tetrahydrofuran ring of aureothin.

Deciphering the biosynthesis of aureothin provided researchers with the opportunity to generate novel analogues of this secondary metabolite, some of which had specific biological activities (Werneburg et al., 2010). Exploiting the relaxed substrate specificity of AurA PKS in terms of the starter unit, and flexibility of the pyrone and tetrahydrofuran rings formation, the authors used mutasynthesis and biotransformation to generate a large number of aureothin analogues. Mutasynthesis is an approach of generating analogues of secondary metabolite by partially disrupting a biosynthetic pathway via genetic engineering while supplying a substrate for the enzyme that performs a biosynthetic step past the one that has been interrupted. By feeding a bacterium having a disrupted biosynthetic pathway with alternative substrates it is possible to obtain a large number of analogues, providing the abovementioned enzyme has relaxed substrate specificity (Maier, 2015; Mu et al., 2022). In biotransformation experiments, a recombinant bacterium is constructed that expresses an enzyme (e.g. prenyltransferase, halogenase etc) that can modify a secondary metabolite which is fed to the culture of such bacterium, yielding new analogues (Li & Heide, 2005).

**FIGURE 5:**
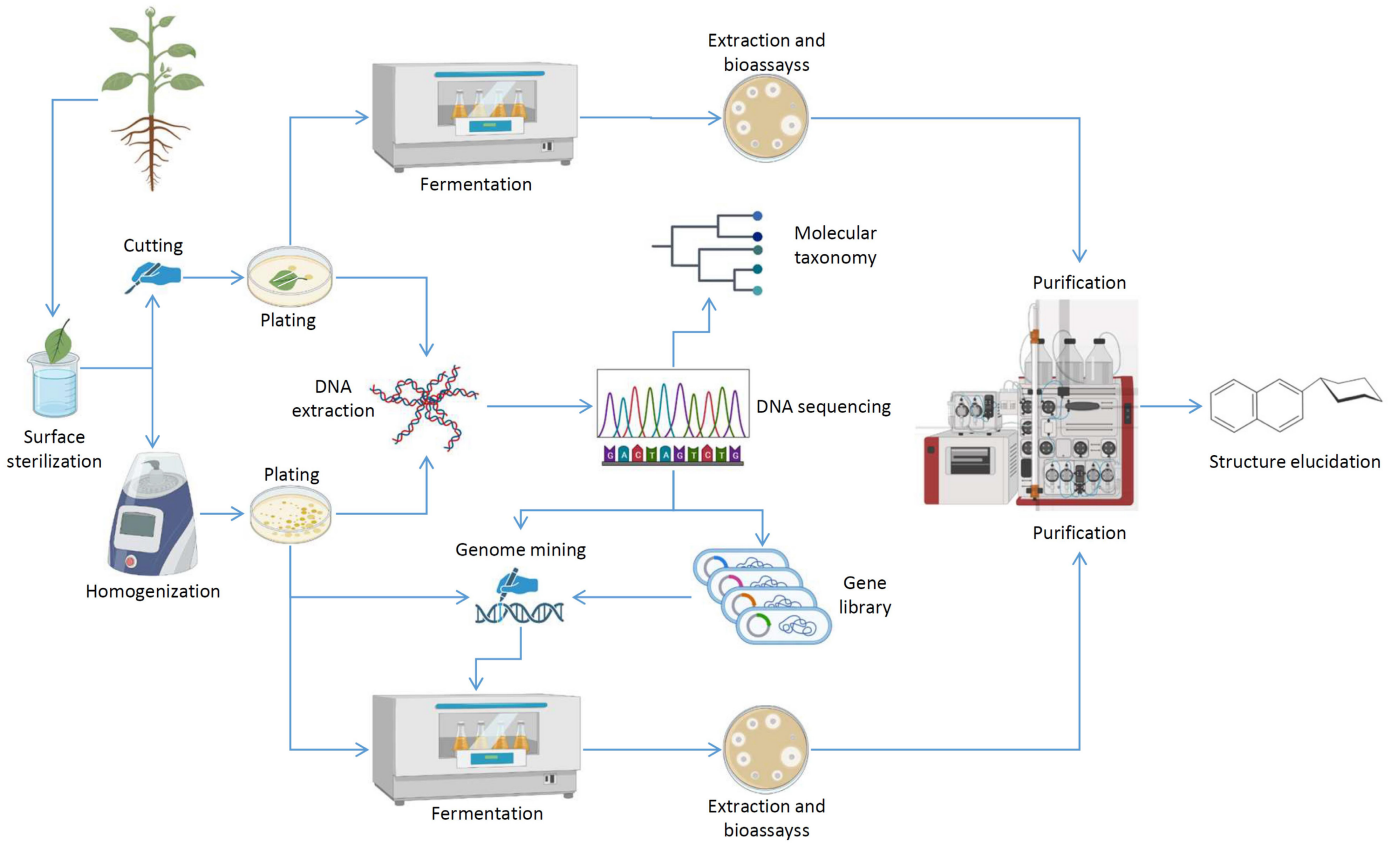
Envisaged workflow for exploration and exploitation of endophytic bacteria for the unravelling of bioactive secondary metabolites that may prove useful for drug discovery. Drawing was created using templates from BioRender.com (2023) (https://app.biorender.com/biorender‐templates) with modifications to the original templates.

Some of the aureoxin analogues generated using these approaches had significantly increased anti‐proliferative activity compared to aureothin, while having much lower cytotoxicity. Other analogues obtained in this way displayed highly selective anti‐fungal activity (see Figure 5). This study provides an important example of how the knowledge of secondary metabolite biosynthesis and the ability to rationally interfere with it can lead to the discovery of new bioactive molecules.

Giving the significant number of bioactive secondary metabolites already identified from endophytic bacteria, and the fact that only a limited number of medicinal plants have been used to isolate endophytes, it is clear that potential for the drug discovery from these organisms is very high. The latter is also becoming evident from the information acquired upon sequencing of the genomes of endophytic bacteria followed by bioinformatics analyses aimed at the identification of diverse BGCs. Although most of these BGCs are not expressed in laboratory conditions, recent advances in metabolic engineering and synthetic biology make it easier to activate them, hence stimulating the production of potentially novel bioactive compounds (Nielsen & Keasling, 2016). At the same time, conventional bioprospecting empowered by the state‐of‐the‐art analytical techniques shall not be forgotten, and can also yield promising bioactive molecules (Maghembe et al., 2020). The next section describes a workflow that can be used to harness the biosynthetic potential of endophytic bacteria, from their isolation to the identification and bioactivity testing of their secondary metabolites.

## PERSPECTIVES OF USING BACTERIAL ENDOPHYTES FOR DRUG DISCOVERY

The workflow for harnessing the secondary metabolite production potential of endophytic bacteria can be envisaged as shown in Figure 5. The whole plant or its parts (e.g. leaves, stem, flowers, roots) are surface‐sterilized to eliminate epiphytic microorganisms using a variety of published methods (Sahu et al., 2022). Considering significant differences between the plants and also their organs in terms of permeability by various sterilizing agents like ethanol, hypochlorite or mercuric chloride, it is necessary to conduct a series of control experiments. Those shall include the use of various surface sterilization strategies followed by the isolation of endophytes to find optimal conditions yielding a maximal number of endophytes and minimal number of contamination by epiphytes. Once the optimal surface sterilization procedure is established, isolation of endophytes may proceed by two methods. A classical one is based on chopping the plant organs into smaller pieces, placing them on the surface of agar nutrient media and incubating for up to several weeks. An alternative method, which could yield a larger number of endophytes, utilizes maceration of sterilized plant organs to obtain fine homogenate, which is plated on agar nutrient media in serial dilutions (Oberhofer et al., 2021). Although this method yields a significantly larger number of endophytes, care must be taken to adjust the extent of homogenization of plant organs to avoid killing the endophytes. With both methods, the choice of agar nutrient medium, temperature and time of incubation are crucial, as they determine the types of bacteria that can be isolated. To further increase the diversity of bacterial endophytes that can be isolated from plants, alternative cultivation methods can be employed. For example, the use of a diffusion chamber described by Bollmann et al. (2007) can be envisaged, although the design of this device must be adapted to plants, for example, via miniaturization. Also, the growth of otherwise ‘uncultivable’ bacteria can be stimulated by signalling molecules such as short peptides (Nichols et al., 2008) or via co‐cultivation with known bacteria‐producing siderophores (D'Onofrio et al., 2010).

Once the bacterial colonies appear on the isolation plates, they must be purified via several sub‐culturing, genomic DNA extracted and used for rapid taxonomic identification at the genus level based on the 16S rRNA gene sequence (Woese et al., 1990). In parallel, whole genome sequencing can be performed, followed by the genome analyses focused on the identification of BGCs and evaluation of the potential of this particular bacterium to produce secondary metabolites.

The first attempt to explore the biosynthetic potential of a bacterium is usually done by cultivating the isolate under various growth conditions. The variables depend on the type of bacterium, its growth rate, optimal growth temperature, aeration and medium composition. The latter must not necessarily support the fast growth of the bacterium, since in most cases production of secondary metabolites is triggered by nutrient limitation (Martín et al., 2011). Typically, bacterial culture is first grown in the medium that ensures the fastest growth, generating the so‐called starting culture. A small portion of this starting culture (typically 5%) is then transferred into the fermentation medium, followed by incubation that may last from 48 h to 20 days. To ensure the capture of a wide variety of secondary metabolites produced, bacterial cultures after fermentation are freeze‐dried and extracted with different solvents, for example, methanol or the mixture of methanol and dichlormethane. The extracts can then be evaporated and dissolved in a small volume of solvent that is compatible with bioassays and analytics (HPLC and LC–MS).

An alternative route to exploring the biosynthetic capacity of endophytic bacteria can be via genome mining, which may employ different strategies (Sekurova et al., 2019). Ultimately, recombinant bacterial strains are generated, represented by either the original bacterium genetically manipulated to activate BGCs potentially specifying biosynthesis of novel metabolites, or heterologous bacterial hosts expressing BGCs cloned from the original isolate or plant‐derived DNA. For the latter, a metagenomics‐based approach can be employed, where total DNA isolated from the plant is sequenced and queried using software such as antiSMASH for the presence of BGCs. In this way, an access to BGCs from as yet uncultivable bacteria can be gained. The BGCs of interest, selected based on their unique features such as gene composition and/or absence of a link to known molecules can be accessed via construction and screening of a genome library. The target BGC (or parts thereof), can then be assembled, for example, using transformation‐associated recombination in yeast in a suitable vector and introduced into a heterologous host (Palazzotto et al., 2019). Methods have also been developed to directly clone entire BGCs from genomic DNA, circumventing the need for the construction of a genomic library (Bian et al., 2012). The constructed recombinant strains are cultivated in a range of media, extracted and analysed by HPLC and LC–MS to compare their secondary metabolomes with those of non‐manipulated strains. Modern approaches for rapid de‐replication of extracts and identification of potentially novel secondary metabolites have been developed, which also assist in the purification of target molecules and elucidation of their structures (van der Hooft et al., 2020). Pairing of BGC‐focused genomics and metabolomics proved to be a powerful tool in the discovery of novel secondary metabolites and de‐replication (reviewed in Avalon et al., 2022). Chemical structure of a secondary metabolite solved via a combination of LC–MS and NMR provides new opportunities for drug discovery, even if the molecule does not exhibit any easy‐to‐test biological activity, such as anti‐microbial or cytotoxic. Modern in silico methods may suggest potential protein targets for the new molecule, thereby pointing toward a bioassay that may confirm the prediction (Chen & Kirchmair, 2020). Application of the workflow described above will most certainly lead to the discovery of novel bioactive secondary metabolites from endophytic bacteria of medicinal plants. Such findings can not only fuel the drug discovery pipeline, but may help us to better understand the ecological roles of endophytes and their influence on the production of secondary metabolites in host plants.

## CONCLUSIONS

All medicinal plants appear to host a large number of taxonomically diverse endophytic bacteria, some of which may provide host plants with certain advantages, both in terms of resistance to biotic/abiotic stress and production of specific secondary metabolites. Moreover, as recent studies on the genomics of endophytic bacteria show, these microorganisms have as yet underexplored capacity to biosynthesize diverse molecules, some of which have specific biological activities. New advances in microbiological techniques focused on the cultivation of bacteria will help to isolate from medicinal plants new species and perhaps new genera of bacteria with unexplored biosynthetic potential. Such isolates can be investigated by state‐of‐the‐art methods encompassing advanced analytics, bio‐ and cheminformatics, a range of sophisticated bioassays etc. Moreover, genome mining, metabolic engineering and synthetic biology approaches can be applied to bacterial endophytes to induce the production of as yet undiscovered secondary metabolites or to boost productivity. Engineering of the biosynthetic pathways for secondary metabolites may yield new analogues with specific biological activities. If implemented in concert, these approaches will open new avenues for drug discovery from endophytic bacteria, and help to better understand their ecological roles.

## FUNDING INFORMATION

The Funding Information section should appear even if no funding information is provided by the author. In that case, the text is: No funding information provided.

## AUTHOR CONTRIBUTIONS

**Sergey B. Zotchev:** Conceptualization (equal); writing – review and editing (equal).

## CONFLICT OF INTEREST STATEMENT

The author declares no conflict of interest.

## References

[mbt214382-bib-0001] Abdelshafy Mohamad, O.A. , Ma, J.B. , Liu, Y.H. , Zhang, D. , Hua, S. , Bhute, S. et al. (2020) Beneficial Endophytic bacterial populations associated with medicinal plant *Thymus vulgaris* alleviate salt stress and confer resistance to *Fusarium oxysporum* . Frontiers in Plant Science, 11, 47. Available from: 10.3389/fpls.2020.00047 32117385 PMC7033553

[mbt214382-bib-0002] Acar, T. , Moreau, S. , Coen, O. , De Meyer, F. , Leroux, O. , Beaumel, M. et al. (2022) Motility‐independent vertical transmission of bacteria in leaf Symbiosis. MBio, 13, e0103322. Available from: 10.1128/mbio.01033-22 36040028 PMC9600174

[mbt214382-bib-0003] Afzal, I. , Shinwari, Z.K. , Sikandar, S. & Shahzad, S. (2019) Plant beneficial endophytic bacteria: mechanisms, diversity, host range and genetic determinants. Microbiological Research, 221, 36–49. Available from: 10.1016/j.micres.2019.02.001 30825940

[mbt214382-bib-0004] Atanasov, A.G. , Waltenberger, B. , Pferschy‐Wenzig, E.M. , Linder, T. , Wawrosch, C. , Uhrin, P. et al. (2015) Discovery and resupply of pharmacologically active plant‐derived natural products: a review. Biotechnology Advances, 33, 1582–1614. Available from: 10.1016/j.biotechadv.2015.08.001 26281720 PMC4748402

[mbt214382-bib-0005] Atanasov, A.G. , Zotchev, S.B. , Dirsch, V.M. , International Natural Product Sciences Taskforce & Supuran, C.T. (2021) Natural products in drug discovery: advances and opportunities. Nature Reviews. Drug Discovery, 20, 200–216. Available from: 10.1038/s41573-020-00114-z 33510482 PMC7841765

[mbt214382-bib-0006] Avalon, N.E. , Murray, A.E. & Baker, B.J. (2022) Integrated Metabolomic‐genomic workflows accelerate microbial natural product discovery. Analytical Chemistry, 94, 11959–11966. Available from: 10.1021/acs.analchem.2c02245 35994737 PMC9453739

[mbt214382-bib-0007] Bekiesch, P. , Oberhofer, M. , Sykora, C. , Urban, E. & Zotchev, S.B. (2021) Piperazic acid containing peptides produced by an endophytic *Streptomyces* sp. isolated from the medicinal plant Atropa belladonna. Natural Product Research, 35, 1090–1096. Available from: 10.1080/14786419.2019.1639174 31303055

[mbt214382-bib-0008] Belcher, M.S. , Mahinthakumar, J. & Keasling, J.D. (2020) New frontiers: harnessing pivotal advances in microbial engineering for the biosynthesis of plant‐derived terpenoids. Current Opinion in Biotechnology, 65, 88–93. Available from: 10.1016/j.copbio.2020.02.001 32155569

[mbt214382-bib-0009] Bian, X. , Huang, F. , Stewart, F.A. , Xia, L. , Zhang, Y. & Müller, R. (2012) Direct cloning, genetic engineering, and heterologous expression of the syringolin biosynthetic gene cluster in E. Coli through red/ET recombineering. ChemBioChem, 13, 1946–1952. Available from: 10.1002/cbic.201200310 22851214

[mbt214382-bib-0010] Bollmann, A. , Lewis, K. & Epstein, S.S. (2007) Incubation of environmental samples in a diffusion chamber increases the diversity of recovered isolates. Applied and Environmental Microbiology, 73, 6386–6390. Available from: 10.1128/AEM.01309-07 17720826 PMC2075052

[mbt214382-bib-0011] Bradley, J.M. , Svistunenko, D.A. , Wilson, M.T. , Hemmings, A.M. , Moore, G.R. & Le Brun, N.E. (2020) Bacterial iron detoxification at the molecular level. The Journal of Biological Chemistry, 295, 17602–17623. Available from: 10.1074/jbc.REV120.007746 33454001 PMC7762939

[mbt214382-bib-0012] Castillo, U.F. , Strobel, G.A. , Ford, E.J. , Hess, W.M. , Porter, H. , Jensen, J.B. et al. (2002) Munumbicins, wide‐spectrum antibiotics produced by *Streptomyces* NRRL 30562, endophytic on *Kennedia nigriscans* . Microbiology, 148, 2675–2685. Available from: 10.1099/00221287-148-9-2675 12213914

[mbt214382-bib-0013] Chavali, A.K. & Rhee, S.Y. (2018) Bioinformatics tools for the identification of gene clusters that biosynthesize specialized metabolites. Briefings in Bioinformatics, 19, 1022–1034. Available from: 10.1093/bib/bbx020 28398567 PMC6171489

[mbt214382-bib-0014] Chen, Y. & Kirchmair, J. (2020) Cheminformatics in natural product‐based drug discovery. Molecular Informatics, 39, e2000171. Available from: 10.1002/minf.202000171 32725781 PMC7757247

[mbt214382-bib-0015] Chiaranunt, P. & White, J.F. (2023) Plant beneficial bacteria and their potential applications in vertical farming systems. Plants, 12, 400. Available from: 10.3390/plants12020400 36679113 PMC9861093

[mbt214382-bib-0016] Compant, S. , Cambon, M.C. , Vacher, C. , Mitter, B. , Samad, A. & Sessitsch, A. (2021) The plant endosphere world ‐ bacterial life within plants. Environmental Microbiology, 23, 1812–1829. Available from: 10.1111/1462-2920.15240 32955144

[mbt214382-bib-0017] Ding, L. & Hertweck, C. (2020) Oxygenated Geosmins and plant‐like Eudesmanes from a bacterial mangrove endophyte. Journal of Natural Products, 83, 2207–2211. Available from: 10.1021/acs.jnatprod.0c00304 32558565

[mbt214382-bib-0018] Ding, L. , Maier, A. , Fiebig, H.H. , Lin, W.H. & Hertweck, C. (2011) A family of multicyclic indolosesquiterpenes from a bacterial endophyte. Organic & Biomolecular Chemistry, 9, 4029–4031. Available from: 10.1039/c1ob05283g 21528153

[mbt214382-bib-0019] Ding, L. , Münch, J. , Goerls, H. , Maier, A. , Fiebig, H.H. , Lin, W.H. et al. (2010) Xiamycin, a pentacyclic indolosesquiterpene with selective anti‐HIV activity from a bacterial mangrove endophyte. Bioorganic & Medicinal Chemistry Letters, 20, 6685–6687. Available from: 10.1016/j.bmcl.2010.09.010 20880706

[mbt214382-bib-0020] D'Onofrio, A. , Crawford, J.M. , Stewart, E.J. , Witt, K. , Gavrish, E. , Epstein, S. et al. (2010) Siderophores from neighboring organisms promote the growth of uncultured bacteria. Chemistry & Biology, 17, 254–264. Available from: 10.1016/j.chembiol.2010.02.010 20338517 PMC2895992

[mbt214382-bib-0021] Ezra, D. , Castillo, U.F. , Strobel, G.A. , Hess, W.M. , Porter, H. , Jensen, J.B. et al. (2004) Coronamycins, peptide antibiotics produced by a verticillate *Streptomyces* sp. (MSU‐2110) endophytic on *Monstera* sp. Microbiology, 150, 785–793. Available from: 10.1099/mic.0.26645-0 15073289

[mbt214382-bib-0022] Fenwick, M.K. , Philmus, B. , Begley, T.P. & Ealick, S.E. (2016) *Burkholderia glumae* ToxA is a dual‐specificity Methyltransferase that catalyzes the last two steps of Toxoflavin biosynthesis. Biochemistry, 55, 2748–2759. Available from: 10.1021/acs.biochem.6b00167 27070241 PMC4870115

[mbt214382-bib-0023] Grininger, M. (2023) Enzymology of assembly line synthesis by modular polyketide synthases. Nature Chemical Biology, 19, 401–415. Available from: 10.1038/s41589-023-01277-7 36914860

[mbt214382-bib-0024] Hardoim, P.R. , van Overbeek, L.S. , Berg, G. , Pirttilä, A.M. , Compant, S. , Campisano, A. et al. (2015) The hidden world within plants: ecological and evolutionary considerations for defining functioning of microbial endophytes. Microbiology and Molecular Biology Reviews, 79, 293–320. Available from: 10.1128/MMBR.00050-14 26136581 PMC4488371

[mbt214382-bib-0025] He, J. & Hertweck, C. (2003) Iteration as programmed event during polyketide assembly; molecular analysis of the aureothin biosynthesis gene cluster. Chemistry & Biology, 10, 1225–1232. Available from: 10.1016/j.chembiol.2003.11.009 14700630

[mbt214382-bib-0026] Huang, X. , Roemer, E. , Sattler, I. , Moellmann, U. , Christner, A. & Grabley, S. (2006) Lydiamycins A‐D: cyclodepsipetides with antimycobacterial properties. Angewandte Chemie (International Ed. in English), 45, 3067–3072. Available from: 10.1002/anie.200503381 16619323

[mbt214382-bib-0027] Igarashi, Y. (2023) Development of a drug discovery approach from microbes with a special focus on isolation sources and taxonomy. The Journal of Antibiotics, 76, 365–383. Available from: 10.1038/s41429-023-00625-y 37188757

[mbt214382-bib-0028] Iqbal, A. , Panta, P.R. , Ontoy, J. , Bruno, J. , Ham, J.H. & Doerrler, W.T. (2021) Chemical or genetic alteration of proton motive force results in loss of virulence of *Burkholderia glumae*, the cause of Rice bacterial panicle blight. Applied and Environmental Microbiology, 87, e0091521. Available from: 10.1128/AEM.00915-21 34260305 PMC8388836

[mbt214382-bib-0029] Jiang, K.L. , Liu, C.M. , Nie, L.T. , Jiang, H.N. , Xu, L. , Zhang, K.Z. et al. (2023) Discovery of toxoflavin, a potent IRE1α inhibitor acting through structure‐dependent oxidative inhibition. Acta Pharmacologica Sinica, 44, 234–243. Available from: 10.1038/s41401-022-00949-9 35840659 PMC9812974

[mbt214382-bib-0030] Jiang, Z.D. , Jensen, P.R. & Fenical, W. (1999) Lobophorins a and B, new antiinflammatory macrolides produced by a tropical marine bacterium. Bioorganic & Medicinal Chemistry Letters, 9, 2003–2006. Available from: 10.1016/s0960-894x(99)00337-6 10450970

[mbt214382-bib-0031] Johnston‐Monje, D. , Gutiérrez, J.P. & Becerra Lopez‐Lavalle, L.A. (2022) Stochastic inoculum, biotic filtering and species‐specific seed transmission shape the rare microbiome of plants. Life, 12, 1372. Available from: 10.3390/life12091372 36143410 PMC9506401

[mbt214382-bib-0032] Kang, M.K. , Kim, J.H. , Liu, M.J. , Jin, C.Z. , Park, D.J. , Kim, J. et al. (2022) New discovery on the nematode activity of aureothin and alloaureothin isolated from endophytic bacteria *Streptomyces* sp. AE170020. Scientific Reports, 12, 3947. Available from: 10.1038/s41598-022-07879-w 35273247 PMC8913828

[mbt214382-bib-0033] Khare, E. , Mishra, J. & Arora, N.K. (2018) Multifaceted interactions between endophytes and plant: developments and prospects. Frontiers in Microbiology, 9, 2732. Available from: 10.3389/fmicb.2018.02732 30498482 PMC6249440

[mbt214382-bib-0034] Kim, N. , Shin, J.C. , Kim, W. , Hwang, B.Y. , Kim, B.S. , Hong, Y.S. et al. (2006) Cytotoxic 6‐alkylsalicylic acids from the endophytic *Streptomyces laceyi* . The Journal of Antibiotics, 59, 797–800. Available from: 10.1038/ja.2006.105 17323647

[mbt214382-bib-0035] Kornfuehrer, T. & Eustáquio, A.S. (2019) Diversification of polyketide structures *via* synthase engineering. MedChemComm, 10, 1256–1272. Available from: 10.1039/c9md00141g 32180918 PMC7053703

[mbt214382-bib-0036] Kumar, A. , Droby, S. , Singh, V.P. , Singh, S.K. & White, J.F. (2020) Entry, colonization, and distribution of endophytic microorganisms in plants. In: Kumar, A. & Radhakrishnan, E.K. (Eds.) Microbial endophytes. Sawston: Woodhead Publishing, pp. 1–33.

[mbt214382-bib-0037] Kuncharoen, N. , Bunbamrung, N. , Intaraudom, C. , Choowong, W. , Thawai, C. , Tanasupawat, S. et al. (2023) Antimalarial and antimicrobial substances isolated from the endophytic actinomycete, *Streptomyces aculeolatus* MS1‐6. Phytochemistry, 207, 113568. Available from: 10.1016/j.phytochem.2022.113568 36565946

[mbt214382-bib-0038] Li, H. , Sun, Y. , Zhang, Q. , Zhu, Y. , Li, S.M. , Li, A. et al. (2015) Elucidating the cyclization cascades in xiamycin biosynthesis by substrate synthesis and enzyme characterizations. Organic Letters, 17, 306–309. Available from: 10.1021/ol503399b 25532029

[mbt214382-bib-0039] Li, H. , Zhang, Q. , Li, S. , Zhu, Y. , Zhang, G. , Zhang, H. et al. (2012) Identification and characterization of xiamycin a and oxiamycin gene cluster reveals an oxidative cyclization strategy tailoring indolosesquiterpene biosynthesis. Journal of the American Chemical Society, 134, 8996–9005. Available from: 10.1021/ja303004g 22591327

[mbt214382-bib-0040] Li, S.M. & Heide, L. (2005) New aminocoumarin antibiotics from genetically engineered *Streptomyces* strains. Current Medicinal Chemistry, 12, 419–427. Available from: 10.2174/0929867053363063 15720250

[mbt214382-bib-0041] Li, X. , Li, Y. , Wang, R. , Wang, Q. & Lu, L. (2019) Toxoflavin produced by *Burkholderia gladioli* from *Lycoris aurea* is a new broad‐Spectrum fungicide. Applied and Environmental Microbiology, 85, e00106‐19. Available from: 10.1128/AEM.00106-19 30824447 PMC6495751

[mbt214382-bib-0042] Liu, H. , Carvalhais, L.C. , Crawford, M. , Singh, E. , Dennis, P. G. , Pieterse, C.M.J. et al. (2017) Inner plant values: diversity, colonization and benefits from endophytic bacteria. Frontiers in Microbiology, 8, 2552. Available from: 10.3389/fmicb.2017.02552 29312235 PMC5742157

[mbt214382-bib-0043] Liu, Y. , Chen, P. , Zhou, M. , Wang, T. , Fang, S. , Shang, X. et al. (2018) Geographic variation in the chemical composition and antioxidant properties of phenolic compounds from *Cyclocarya paliurus* (Batal) Iljinskaja leaves. Molecules, 23, 2440. Available from: 10.3390/molecules23102440 30249997 PMC6222593

[mbt214382-bib-0044] Ludwig‐Müller, J. (2015) Plants and endophytes: equal partners in secondary metabolite production. Biotechnology Letters, 37, 1325–1334. Available from: 10.1007/s10529-015-1814-4 25792513

[mbt214382-bib-0045] Luo, M. , Tang, L. , Dong, Y. , Huang, H. , Deng, Z. & Sun, Y. (2021) Antibacterial natural products lobophorin L and M from the marine‐derived *Streptomyces* sp. 4506. Natural Product Research, 35, 5581–5587. Available from: 10.1080/14786419.2020.1797730 32713197

[mbt214382-bib-0046] Maghembe, R. , Damian, D. , Makaranga, A. , Nyandoro, S.S. , Lyantagaye, S.L. , Kusari, S. et al. (2020) Omics for bioprospecting and drug discovery from bacteria and microalgae. Antibiotics, 9, 229. Available from: 10.3390/antibiotics9050229 32375367 PMC7277505

[mbt214382-bib-0047] Maier, M.E. (2015) Design and synthesis of analogues of natural products. Organic & Biomolecular Chemistry, 13, 5302–5343. Available from: 10.1039/c5ob00169b 25829247

[mbt214382-bib-0048] Martín, J.F. , Sola‐Landa, A. , Santos‐Beneit, F. , Fernández‐Martínez, L.T. , Prieto, C. & Rodríguez‐García, A. (2011) Cross‐talk of global nutritional regulators in the control of primary and secondary metabolism in *Streptomyces* . Microbial Biotechnology, 4, 165–174. Available from: 10.1111/j.1751-7915.2010.00235.x 21342462 PMC3818857

[mbt214382-bib-0049] Meng, Z. , Yu, H. , Li, L. , Tao, W. , Chen, H. , Wan, M. et al. (2015) Total synthesis and antiviral activity of indolosesquiterpenoids from the xiamycin and oridamycin families. Nature Communications, 6, 6096. Available from: 10.1038/ncomms7096 PMC4347019 25648883

[mbt214382-bib-0050] Meurer, G. , Gerlitz, M. , Wendt‐Pienkowski, E. , Vining, L.C. , Rohr, J. & Hutchinson, C.R. (1997) Iterative type II polyketide synthases, cyclases and ketoreductases exhibit context‐dependent behavior in the biosynthesis of linear and angular decapolyketides. Chemistry & Biology, 4, 433–443. Available from: 10.1016/s1074-5521(97)90195-2 9224566

[mbt214382-bib-0051] Mu, N. , Guo, H. , Zhang, E. , Yin, Y. , Wang, W. , Chen, D. et al. (2022) Mutasynthesis generates antibacterial Benzothiophenic‐containing Nosiheptide analogues. Journal of Natural Products, 85, 2274–2281. Available from: 10.1021/acs.jnatprod.2c00273 36122372

[mbt214382-bib-0052] Nichols, D. , Lewis, K. , Orjala, J. , Mo, S. , Ortenberg, R. , O'Connor, P. et al. (2008) Short peptide induces an "uncultivable" microorganism to grow in vitro. Applied and Environmental Microbiology, 74, 4889–4897. Available from: 10.1128/AEM.00393-08 18515474 PMC2519364

[mbt214382-bib-0053] Nielsen, J. & Keasling, J.D. (2016) Engineering cellular metabolism. Cell, 164, 1185–1197. Available from: 10.1016/j.cell.2016.02.004 26967285

[mbt214382-bib-0054] Oberhofer, M. , Wackerlig, J. , Zehl, M. , Büyük, H. , Cao, J.J. , Prado‐Roller, A. et al. (2021) Endophytic *Akanthomyces* sp. LN303 from edelweiss produces Emestrin and two new 2‐Hydroxy‐4 Pyridone alkaloids. ACS Omega, 6, 2184–2191. Available from: 10.1021/acsomega.0c05472 33521458 PMC7841945

[mbt214382-bib-0055] Otoguro, K. , Ishiyama, A. , Namatame, M. , Nishihara, A. , Furusawa, T. , Masuma, R. et al. (2008) Selective and potent in vitro antitrypanosomal activities of ten microbial metabolites. The Journal of Antibiotics, 61, 372–378. Available from: 10.1038/ja.2008.52 18667785

[mbt214382-bib-0056] Oukala, N. , Aissat, K. & Pastor, V. (2021) Bacterial endophytes: the hidden actor in plant immune responses against biotic stress. Plants, 10, 1012. Available from: 10.3390/plants10051012 34069509 PMC8161118

[mbt214382-bib-0057] Palazzotto, E. , Tong, Y. , Lee, S.Y. & Weber, T. (2019) Synthetic biology and metabolic engineering of actinomycetes for natural product discovery. Biotechnology Advances, 37, 107366. Available from: 10.1016/j.biotechadv.2019.03.005 30853630

[mbt214382-bib-0058] Pandey, S.S. , Singh, S. , Babu, C.S. , Shanker, K. , Srivastava, N.K. & Kalra, A. (2016) Endophytes of opium poppy differentially modulate host plant productivity and genes for the biosynthetic pathway of benzylisoquinoline alkaloids. Planta, 243, 1097–1114. Available from: 10.1007/s00425-016-2467-9 26794966

[mbt214382-bib-0059] Philmus, B. , Shaffer, B.T. , Kidarsa, T.A. , Yan, Q. , Raaijmakers, J.M. , Begley, T.P. et al. (2015) Investigations into the biosynthesis, regulation, and self‐resistance of Toxoflavin in *pseudomonas protegens* Pf‐5. Chembiochem, 16, 1782–9170. Available from: 10.1002/cbic.201500247 26077901

[mbt214382-bib-0060] Puja, H. , Mislin, G.L.A. & Rigouin, C. (2023) Engineering Siderophore biosynthesis and regulation pathways to increase diversity and availability. Biomolecules, 13, 959. Available from: 10.3390/biom13060959 37371539 PMC10296737

[mbt214382-bib-0061] Sahu, P.K. , Tilgam, J. , Mishra, S. , Hamid, S. , Gupta, A.K.J. , Verma, S.K. et al. (2022) Surface sterilization for isolation of endophytes: ensuring what (not) to grow. Journal of Basic Microbiology, 62, 647–668. Available from: 10.1002/jobm.202100462 35020220

[mbt214382-bib-0062] Sasse, J. , Martinoia, E. & Northen, T. (2018) Feed your friends: do Plant exudates shape the root microbiome? Trends in Plant Science, 23, 25–41. Available from: 10.1016/j.tplants.2017.09.003 29050989

[mbt214382-bib-0063] Scherlach, K. & Hertweck, C. (2020) Chemical mediators at the bacterial‐fungal Interface. Annual Review of Microbiology, 74, 267–290. Available from: 10.1146/annurev-micro-012420-081224 32660387

[mbt214382-bib-0064] Sekurova, O.N. , Schneider, O. & Zotchev, S.B. (2019) Novel bioactive natural products from bacteria via bioprospecting, genome mining and metabolic engineering. Microbial Biotechnology, 12, 828–844. Available from: 10.1111/1751-7915.13398 30834674 PMC6680616

[mbt214382-bib-0065] Shang, N.N. , Zhang, Z. , Huang, J.P. , Wang, L. , Luo, J. , Yang, J. et al. (2018) Glycosylated piericidins from an endophytic streptomyces with cytotoxicity and antimicrobial activity. The Journal of Antibiotics, 71, 672–676. Available from: 10.1038/s41429-018-0051-1 29651143

[mbt214382-bib-0066] Song, K. , Li, W. , Zhao, Z. , Li, H. , Liu, Y. , Zhao, G. et al. (2023) Heterologous reconstitution of Toxoflavin biosynthesis reveals key pathway intermediates and a cofactor‐independent oxidase. Organic Letters, 25, 2918–2922. Available from: 10.1021/acs.orglett.3c01000 37074364

[mbt214382-bib-0067] Spagnolo, F. , Trujillo, M. & Dennehy, J.J. (2021) Why do antibiotics exist? mBio, 12, e0196621. Available from: 10.1128/mBio.01966-21 34872345 PMC8649755

[mbt214382-bib-0068] Taghinasab, M. & Jabaji, S. (2020) Cannabis microbiome and the role of endophytes in modulating the production of secondary metabolites: an overview. Microorganisms, 8, 355. Available from: 10.3390/microorganisms8030355 32131457 PMC7143057

[mbt214382-bib-0069] Tsalgatidou, P.C. , Thomloudi, E.E. , Baira, E. , Papadimitriou, K. , Skagia, A. , Venieraki, A. et al. (2022) Integrated genomic and Metabolomic analysis illuminates key secreted metabolites produced by the novel endophyte *bacillus halotolerans* Cal.L.30 involved in diverse biological control activities. Microorganisms, 10, 399. Available from: 10.3390/microorganisms10020399 35208854 PMC8877463

[mbt214382-bib-0070] Uchida, R. , Tomoda, H. & Omura, S. (2006) Biosynthesis of sespendole. The Journal of Antibiotics, 59, 298–302. Available from: 10.1038/ja.2006.42 16883780

[mbt214382-bib-0071] Um, S. , Lee, J. & Kim, S.H. (2022) Lobophorin producing Endophytic *Streptomyces olivaceus* JB1 associated with *Maesa japonica* (Thunb.) Moritzi & Zoll. Frontiers in Microbiology, 13, 881253. Available from: 10.3389/fmicb.2022.881253 35572656 PMC9100408

[mbt214382-bib-0072] van Damme, P.A. , Johannes, A.G. , Cox, H.C. & Berends, W. (1960) On toxoflavin, the yellow poison of *pseudomonas cocovenenans* . Recueil Des Travaux Chimiques Des Pays‐Bas, 79, 255–267.

[mbt214382-bib-0073] van der Hooft, J.J.J. , Mohimani, H. , Bauermeister, A. , Dorrestein, P.C. , Duncan, K.R. & Medema, M.H. (2020) Linking genomics and metabolomics to chart specialized metabolic diversity. Chemical Society Reviews, 49, 3297–3314. Available from: 10.1039/d0cs00162g 32393943

[mbt214382-bib-0074] Wang, W. , Kim, S. , Vu, T.H.N. , Quach, N.T. , Oh, E. , Park, K.H. et al. (2023) Bioactive Piperazic acid‐bearing Cyclodepsipeptides, Lydiamycins E‐H, from an Endophytic *Streptomyces* sp. associated with *Cinnamomum cassia* . Journal of Natural Products, 86, 751–758. Available from: 10.1021/acs.jnatprod.2c00902 Epub ahead of print. 36812487

[mbt214382-bib-0075] Washizu, F. , Umezawa, H. & Sugiyama, N. (1954) Chemical studies on a toxic product of *Streptomyces thioluteus*, aureothin. The Journal of Antibiotics, 7, 60. 13174453

[mbt214382-bib-0076] Wassermann, B. , Abdelfattah, A. , Wicaksono, W.A. , Kusstatscher, P. , Müller, H. , Cernava, T. et al. (2022) The *Brassica napus* seed microbiota is cultivar‐specific and transmitted via paternal breeding lines. Microbial Biotechnology, 15, 2379–2390. Available from: 10.1111/1751-7915.14077 35593114 PMC9437892

[mbt214382-bib-0077] Wawrosch, C. & Zotchev, S.B. (2021) Production of bioactive plant secondary metabolites through in vitro technologies‐status and outlook. Applied Microbiology and Biotechnology, 105, 6649–6668. Available from: 10.1007/s00253-021-11539-w 34468803 PMC8408309

[mbt214382-bib-0078] Wei, R.B. , Xi, T. , Li, J. , Wang, P. , Li, F.C. , Lin, Y.C. et al. (2011) Lobophorin C and D, new kijanimicin derivatives from a marine sponge‐associated actinomycetal strain AZS17. Marine Drugs, 9, 359–368. Available from: 10.3390/md9030359 21556165 PMC3083656

[mbt214382-bib-0079] Werneburg, M. , Busch, B. , He, J. , Richter, M.E.A. , Xiang, L. , Moore, B.S. et al. (2010) Exploiting enzymatic promiscuity to engineer a focused library of highly selective antifungal and antiproliferative aureothin analogues. Journal of the American Chemical Society, 132, 10407–10413. Available from: 10.1021/ja102751h 20662518 PMC2925430

[mbt214382-bib-0080] Wink, M. (2003) Evolution of secondary metabolites from an ecological and molecular phylogenetic perspective. Phytochemistry, 64, 3–19. Available from: 10.1016/s0031-9422(03)00300-5 12946402

[mbt214382-bib-0081] Woese, C.R. , Kandler, O. & Wheelis, M.L. (1990) Towards a natural system of organisms: proposal for the domains archaea, bacteria, and Eucarya. Proceedings of the National Academy of Sciences of the USA, 7, 4576–4579. Available from: 10.1073/pnas.87.12.4576 PMC54159 2112744

[mbt214382-bib-0082] Xu, Z. , Baunach, M. , Ding, L. & Hertweck, C. (2012) Bacterial synthesis of diverse indole terpene alkaloids by an unparalleled cyclization sequence. Angewandte Chemie International Edition, 51, 10293–10297. Available from: 10.1002/anie.201204087 22968942

[mbt214382-bib-0083] Yang, A. , Zeng, S. , Yu, L. , He, M. , Yang, Y. , Zhao, X. et al. (2018) Characterization and antifungal activity against *Pestalotiopsis* of a fusaricidin‐type compound produced by *Paenibacillus polymyxa* Y‐1. Pesticide Biochemistry and Physiology, 147, 67–74. Available from: 10.1016/j.pestbp.2017.08.012 29933995

[mbt214382-bib-0084] Yang, Y.H. , Yang, D.S. , Li, G.H. , Liu, R. , Huang, X.W. , Zhang, K.Q. et al. (2018) New secondary metabolites from an engineering mutant of endophytic *Streptomyces* sp. CS. Fitoterapia, 130, 17–25. Available from: 10.1016/j.fitote.2018.07.019 30076887

[mbt214382-bib-0085] Yin, D.D. , Yang, M. , Wang, Y.L. , Yin, D.K. , Liu, H.K. , Zhou, M. et al. (2018) High tetramethylpyrazine production by the endophytic bacterial *Bacillus subtilis* isolated from the traditional medicinal plant *Ligusticum chuanxiong* Hort. AMB Express, 8, 193. Available from: 10.1186/s13568-018-0721-1 30564983 PMC6298913

[mbt214382-bib-0086] Zhang, C. , Fan, L. , Fan, S. , Wang, J. , Luo, T. , Tang, Y. et al. (2019) *Cinnamomum cassia* Presl: a review of its traditional uses, phytochemistry, pharmacology and toxicology. Molecules, 24, 3473. Available from: 10.3390/molecules24193473 31557828 PMC6804248

[mbt214382-bib-0087] Zhang, J. , Zang, S. , Bai, B. & Fan, S. (2019) Isolation and screening for limonin‐producing endophytic bacteria from *Citrus maxima* (Burm.) Merr. cv. Shatian Yu. Biotechnology and Applied Biochemistry, 66, 192–201. Available from: 10.1002/bab.1721 30578642

[mbt214382-bib-0088] Zhang, Q. , Li, H. , Yu, L. , Sun, Y. , Zhu, Y. , Zhu, H. et al. (2017) Characterization of the flavoenzyme XiaK as an *N*‐hydroxylase and implications in indolosesquiterpene diversification. Chemical Science, 8, 5067–5077. Available from: 10.1039/c7sc01182b 28970893 PMC5613243

[mbt214382-bib-0089] Zhao, H. , Yang, A. , Liu, J. , Bao, S. , Peng, R. , Hu, Y. et al. (2020) Chartspiroton, a tetracyclic Spiro‐naphthoquinone derivative from a medicinal plant Endophytic *Streptomyces* . Organic Letters, 22, 3739–3743. Available from: 10.1021/acs.orglett.0c00696 32186890

[mbt214382-bib-0090] Zhao, H. , Yang, A. , Zhang, N. , Li, S. , Yuan, T. , Ding, N. et al. (2020) Insecticidal Endostemonines A‐J produced by Endophytic *Streptomyces* from *Stemona sessilifolia* . Journal of Agricultural and Food Chemistry, 68, 1588–1595. Available from: 10.1021/acs.jafc.9b06755 31994388

[mbt214382-bib-0091] Zhou, J.Y. , Sun, K. , Chen, F. , Yuan, J. , Li, X. & Dai, C.C. (2018) Endophytic *pseudomonas* induces metabolic flux changes that enhance medicinal sesquiterpenoid accumulation in *Atractylodes lancea* . Plant Physiology and Biochemistry, 130, 473–481. Available from: 10.1016/j.plaphy.2018.07.016 30081324

[mbt214382-bib-0092] Zhu, Y. , Kong, Y. , Hong, Y. , Zhang, L. , Li, S. , Hou, S. et al. (2022) Huoshanmycins A–C, new polyketide dimers produced by Endophytic *Streptomyces* sp. HS‐3‐L‐1 from *Dendrobium huoshanense* . Frontiers in Chemistry, 9, 807508. Available from: 10.3389/fchem.2021.807508 35237566 PMC8883461

